# The complete mitochondrial genome sequence of *Xenocypris fangi*

**DOI:** 10.1080/23802359.2021.1903361

**Published:** 2021-03-24

**Authors:** Liu Nannan, Gu Haoran, Cheng Xinkai, Wang Yuanfu, Wang Zhijian

**Affiliations:** Key Laboratory of Freshwater Fish and Development (Ministry of Education), Key Laboratory of Aquatic Science of Chongqing, School of life Sciences, Southwest University, Chongqing, China

**Keywords:** *Xenocypris fangi*, mitochondrial genome, phylogeny

## Abstract

*Xenocypris fangi* Tchang (1930) is an endemic species in China, which is mainly distributed in the Jialing River and its tributaries. In this study, the complete mitochondrial genome was sequenced. Its length is 16,619 bp, containing 22 tRNAs, 2 rRNAs, and 13 protein-coding genes (PCGs). The mitochondrial genome has common characteristics with other teleosts in the genetic arrangement. The phylogenetic tree shows that *X. fangi* is closely related to *Distoechodon tumirostris*.

## Introduction

*Xenocypris fangi* belongs to the Cypriniformes, Cyprinidae, Xenocyprinae, and *Xenocypris Günther*. It is an endemic species with fast growth rate and high economic value in China. The complete mitochondrial genome plays an important role in species evolution and phylogenetic analysis. However, the complete mitochondrial genome of *X. fangi* has not been reported. Therefore, in this study, the whole mitochondrial genome of *X. fangi* was sequenced and analyzed. The samples were collected from the Jialing River (Guangyuan, Sichuang, China; 32°61′07′′N, 105°82′95′′E), and a specimen was deposited at Southwest University Museum of Zoology under the voucher number Y1002201707170015. Meanwhile, the phylogenetic tree was constructed by comparing the mitochondrial genomes with other species of the Xenocyprinae which can provide a basis for further genetic research on fishes in this subfamily.

## Material and method

The DNA extraction kit (DC102 FastPure CellTissue DNA Isolation Mini Kit) was used to extract the genomic DNA from *X. fangi*’s pelvic fin. The paired-end (2 × 150 bp) library was constructed by the Illumina Hiseq 5000. The mitochondrial genome was assembled by NOVOPlasty (https://github.com/ndierckx/NOVOPlasty) with *Xenocypris davidi* (GenBank: KF039718) (Liu 2014) as the initial reference, and online tool MITOS (http://mitos2.bioinf.uni-leipzig.de/index.py) was used to annotate the mitochondrial genome. The ML phylogenetic tree (Best-fit model: TIM2 + F + G4 chosen according to BIC) was constructed by IQ-TREE with a default setting (Trifinopoulos et al. 2016).

## Results

The length of complete mitochondrial genome is 16,619 bp, including 22 transfer RNAs (tRNAs) with a total length of 1566 bp, 2 ribosomal RNAs (rRNAs) with a total length of 2606 bp, and 13 protein-coding genes (PCGs). The PCGs contain *COX1*, *COX2*, *ATP8*, *ATP6*, *COX3*, *ND3*, *ND1*, *ND5*, *ND4*, *ND4L*, *ND6*, *CYTB*, and *ND2*. The arrangement of all genes is the same as most teleosts (Chen et al. 2013; Liu 2014; Asem et al. 2018). The *ND6* and eight tRNAs are encoded on the light (L) strain, and the other genes are encoded on the heavy (H) strain. Among PCGs, the start codon of *COX1* is GTG, the start codon of other PCGs are ATG; The stop codon of *ND2*, *ATP8*, and *ND3* are TAG and the rest mostly are TAA as the stop codon. The incomplete stop codon T is found in *COX2* and *CYTB*, and the incomplete stop codon TA is found in *ND4*. The nucleotide composition of the whole mitochondrial genome of *X. fangi* includes 31.25% A, 25.3% T, 16.18% G, and 27.28% C.

**Figure 1.**
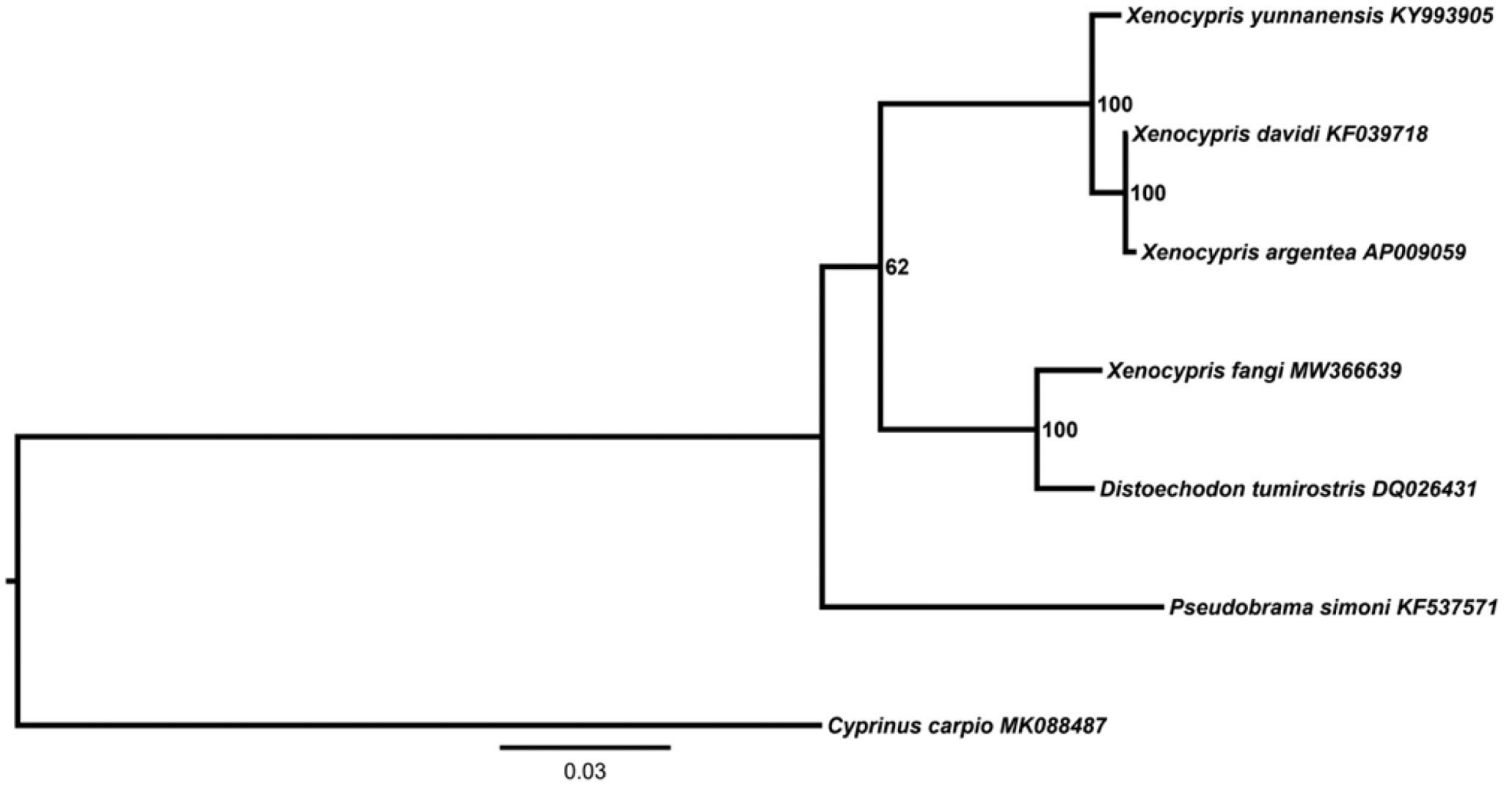
Phylogenetic tree showing the relationship among *X. fangi* and 5 other species of Xenocyprinae fishes. Numbers in front of each node indicates the bootstrap support value. The GenBank accession numbers are indicated on the right side of species names.

In order to study the phylogenetic relationship between *X. fangi* and five species of Xenocyprinae fishes (*X. davidi*, *Xenocypris yunnanensis*, *Xenocypris argentea*, *Distoechodon tumirostris*, *Pseudobrama simoni*), a maximum-likelihood (ML) phylogenetic tree was constructed. The results showed that *D. tumirostris* and *X. fangi* were clustered in a clade, suggesting that the two were closely related (Figure 1).

## Data Availability

The genome sequence data that support the findings of this study are openly available in GenBank of NCBI at (https://www.ncbi.nlm.nih.gov/) under the accession no. MW366639.
